# Do government expenditure and financial development impede environmental degradation in Venezuela?

**DOI:** 10.1371/journal.pone.0210255

**Published:** 2019-01-10

**Authors:** Alotaish Mohammed Saud M., Ping Guo, Ihtisham ul Haq, Guoqin Pan, Alam Khan

**Affiliations:** 1 School of Economics and Trade, Hunan University, Changsha, Hunan, China; 2 Department of Economics, Kohat University of Science and Technology, Kohat, KP, Pakistan; Shandong University of Science and Technology, CHINA

## Abstract

Environmental degradation is causing global warming, which is of the utmost concern to both physical and social scientists. A number of potential determinants of environmental degradation are analysed in the literature. This study examines the role of government expenditure and financial development in environmental degradation in the context of the environmental Kuznets curve (EKC) hypothesis for the Venezuelan economy. Time series data have been analysed for this purpose. The long-term relationship between the variables in this study is established through a bounds test in the presence of an unknown structural break. The results of this study confirm the EKC hypothesis. It is found that energy use is harming the quality of the environment not only in the long run but also in the short run. This study finds a positive impact of government expenditure on environmental degradation, which indicates that the Venezuelan government is not taking its expenditure for a sustainable environment into account. Moreover, this study finds that financial development is hindering environmental degradation. This means that financial institutions in Venezuela can help to develop the concept of sustainable energy in the country and the Venezuelan government can reduce carbon emissions through financial development.

## Introduction

Environmental degradation is causing global warming, which is of the utmost concern to both physical and social scientists as it has adverse effects on human beings [1]. Today, not a single region or country is solely responsible for global warming; therefore, each region or country should take steps to improve the quality of the environment by lessening the emissions of greenhouse gases, which are a cause of global warming. However, among the greenhouse gases, carbon emissions contribute the most to global warming. This is the reason that carbon emissions are used as a proxy for environmental degradation in a number of empirical studies [2, 3, 4].

In the literature, the most prominent determinant of environmental degradation is economic activity. More economic activity means an increase in the income level of the masses, but it comes at the expense of the depletion of natural resources and thus, the degradation of environmental quality. The pioneering work of Grossman and Krueger [5] on the relationship between income level and environmental degradation is based on the Kuznets [6] study that identified an inverted U-shaped relationship between income growth and income inequality. The non-linear relationship between income level and environmental degradation is known as the environmental Kuznets curve (EKC). The EKC hypothesis postulates that, during the initial period of economic development, the environment degrades with the increase in economic growth as resources are depleted. This means that an increase in income level is followed by environmental degradation. However, in the later stages of economic development, the quality of the environment improves as income levels increase and people and governments raise the issues related to the environment and public health. Thus, the EKC hypothesis describes an inverted U-shaped relationship between income level and environmental degradation. A number of research studies affirmed the EKC hypothesis [2, 7–8]. However, there are studies that did not confirm the EKC hypothesis [3–4, 9–11].

Energy use is a major source of economic growth, but in most countries, the share of renewable energy is less than that of non-renewable energy. Energy use aggravates environmental degradation because energy produces emissions and hence, pollutes the environment. This is the reason that energy use attracts attention as an environmental degradation factor in the context of the EKC hypothesis. In most cases, the empirical studies found a detrimental effect of energy on the environment [3–4, 7–8, 10–11]. Similarly, in the literature that is not focused on energy use, other factors that have been shown to influence environmental pollution are population growth, energy prices, trade and consumer behaviour [5, 12–16]. Similarly, financial development is also analysed in the environmental degradation-growth nexus. The literature related to financial development and environmental quality provides mix results. Some studies, such as Tamazian et al. [17] and Jalil and Feridun [18], confirm a negative relationship between financial development and environmental degradation and deduce that financial development lowers the carbon emissions and thereby, reduces environmental degradation. On the other hand, some empirical studies, such as Sadorsky [19] and Zhang [20], find a positive relationship between financial development and environmental degradation. These studies argue that a sound financial development reduces financial costs and increases the number of financing channels. It also diversifies financial risk. As a result, financial development stimulates the industrialization of the economy and pollutes the environment as emissions increase. Additionally, sound financial development attracts foreign investors to the financial market who increase the financial assistance to the corporate sector, which leads to more emissions. From the consumption point of view, sound financial development gives more incentive and opportunities for consumers to finance consumer durable goods that further intensify emissions.

Calbick and Gunton [21] assert that, in developed countries, policy factors are the most important element for measuring variation in the amount of environmental pollution. Thus, government expenditure is also attracting researchers who wish to determine its effect on environmental quality. The studies of Bernauer and Koubi [22], Frederik and Lundstrom [23], Lopez et al. [24], Halkos and Paizanos [25], Lopez and Palacios [26] and Islam and Lopez [27] show that government expenditure is one of the important factors for determining environmental quality. These empirical studies are supported by the theoretical works of Heyes [28], Lawn [29], and Sim [30]. Even though the environment is considered to be a basic element of society, it is neglected in regard to government expenditure because little urgency has been placed on environmental protection. In general, environmental protection is not the priority and goal of most countries’ economies. Moreover, there have been no generalized rules and regulations that classify the role of government expenditure in environmental protection. Fredrick and Lundstrom [23] opined that if environmental quality is considered as a luxury public good, then environmental quality may be given due concern after necessary public goods are supplied. This literature study gives the impression that environmental quality will be given due concern in those economies that have a large amount of government expenditure. Similarly, an increase in government expenditure will improve environmental regulations, which will further improve and enhance the institutions that work for the betterment of environmental quality [31]. The control of pollution and the improvement of environmental quality depends on the magnitude of the share of government expenditure on the environment and on regulations related to environmental quality. Galinato and Islam [32] argue that rules and regulations are much firmer in democratic governments than in dictatorships. This study gives the impression that democratic regimes are much more involved in the rules and regulations of environmental quality. Moreover, the government may adopt policies that can generate less pollution, such as investing in the transportation sector, which may lead to reduced air pollution compared to the use of private transportation [24, 27]. In contrast to the mentioned case studies, the study of Bernauer et al. [33] finds that increases in government expenditure do not necessarily improve the quality of the environment. Instead, the study concludes that government expenditure leads to the deterioration of environmental quality, while Frederik and Lundstrom [23] conclude that environmental quality is related to economic freedom subject to the size of the government. The higher the level of the economic freedom, along with a smaller government, the smaller the effects of pollution compared to low levels of economic freedom along with a larger government.

The prime objective of this study is to examine the role of government expenditure and financial development in the environmental degradation of Venezuela. In addition, this study is going to test the validity of the EKC hypothesis in Venezuela. This study identifies the research gap that no study has considered government expenditure along with financial development as determinants of environmental degradation. Finally, this study also adds the case of Venezuela to the existing literature because this is the first study to examine the effects of government expenditure and financial development on environmental degradation in the EKC context in Venezuela.

## Material and methods

The empirical model of the study is explained as follows. Recently, numerous empirical studies examined energy as the determinant of environmental degradation in the EKC context [3–4, 34]. Similarly, financial development, along with energy, is identified as a determinant of environmental degradation in the empirical studies of Tamazian et al. [17] and Shahbaz et al. [35]. Government expenditure is considered to be a determinant of environmental degradation in a number of studies [22–27, 36]. Thus, this study developed the following model:

CEP=f(PC,PCS,EU,FD,GX) (1)

The model of the study in the logarithmic form can be written as:

$$ln{CEP}_{t}=\alpha_{0}+\alpha_{pc}lnPC_{t}+\alpha_{pcs}{lnPCS}_{t}+\alpha_{EU}lnEU_{t}+\alpha_{FD}{lnFD}_{t}+\alpha_{GE}lnGX_{t}+\epsilon_{t}$$ (2)

*CEP*, *PC*, *PCS*, *EU*, *FD*, and *GX* represent carbon emissions per capita, income level, squares of the income level, energy use, financial development, and government expenditure, respectively. The EKC hypothesis will only be validated if the coefficient of income and its squares are significantly positive and negative, respectively. We expect a positive effect of energy on environmental degradation. Financial development and government expenditure may carry a positive or a negative sign depending on their impact on environmental degradation in Venezuela.

Data on all variables are collected from the World Development Indicators online database [37]. This study used carbon emissions (metric tons per capita) as a proxy for environmental degradation. Real gross domestic product (GDP) per capita is used as a proxy for income level. Energy consumption per capita is used for energy use. The ratio of private domestic credit to GDP is used as a proxy for financial development, whereas the ratio of government expenditure to GDP is used as a proxy for government expenditure. Time series data is analysed on Eviews-9 over the period from 1971 to 2013. Before the analysis, the data was checked for time series properties in the presence of an unknown structural break. This study will employ the Zivot and Andrew structural break unit root [38] for this purpose. An Autoregressive Distributed Lag Model (ARDL) will be employed for cointegration and to obtain long run and short run estimates. This model is an amalgamation of the autoregressive and distributed lag models developed by Pesaran et al. [39]. There are multiple advantages of this technique over other techniques of cointegration. It not only avoids the problem of endogeneity since it distinguishes between the dependent and independent variables but also determines the long run and short run estimates. Its estimates are unbiased and efficient because it avoids the problem of autocorrelation and endogeneity [39]. It is worth mentioning that the advantage of this model over other techniques of cointegration is that it can be applied irrespective of the order of integration of the variables. Thus, it is not important whether variables are integrated as an order of I (0) or I (1) as in other techniques, such as the Johansen cointegration technique for which the variables have to be integrated in the same order. However, the model cannot be applied to a higher order of integration than I (1). Similarly, it does not matter whether its order of integration is I (0) or I (1) or a combination of both. The F-statistic values have to be greater than the upper bound critical values to conclude cointegration [39]. The unrestricted error correction models (UECM) are expressed in equation form in this study and are presented in Eqs 3 to 8, in which β_i_ and γ_i_ capture short run and long run estimates, respectively. The cointegration results will be obtained from analysing all equations, while long run and short run estimates will be obtained from Eq 3 only. The optimal lag in these UECM will be determined according to the Akaike information criteria.

$$\Delta ln{CE}_{t}=\beta_{0}+\sum_{i=1}^{q1}\beta_{1}{\Delta lnCE}_{t-i}+\sum_{i=0}^{q2}\beta_{2}{\Delta lnPC}_{t-i}+\sum_{i=0}^{q3}\beta_{3}{\Delta lnPCS}_{t-i}+\sum_{i=0}^{q4}\beta_{4}{\Delta lnEU}_{t-i}+\sum_{i=0}^{q5}\beta_{5}{\Delta lnFD}_{t-i}+\sum_{i=0}^{q4}\beta_{6}\Delta {lnGX}_{t-i}+\gamma_{1}ln{CE}_{t-1}+\gamma_{2}ln{PC}_{t-1}+\gamma_{2}ln{PCS}_{t-1}+\gamma_{3}ln{EU}_{t-1}+\gamma_{4}ln{FD}_{t-1}+\gamma_{5}ln{GX}_{t-1}+\gamma_{D}Dummy+\mu_{t}$$ (3)

$$\Delta ln{PC}_{t}=\beta_{0}+\sum_{i=0}^{q1}\beta_{1}{\Delta lnCE}_{t-i}+\sum_{i=1}^{q2}\beta_{2}{\Delta lnPC}_{t-i}+\sum_{i=0}^{q3}\beta_{3}{\Delta lnPCS}_{t-i}+\sum_{i=0}^{q4}\beta_{4}{\Delta lnEU}_{t-i}+\sum_{i=0}^{q5}\beta_{5}{\Delta lnFD}_{t-i}+\sum_{i=0}^{q4}\beta_{6}\Delta {lnGX}_{t-i}+\gamma_{1}ln{CE}_{t-1}+\gamma_{2}ln{PC}_{t-1}+\gamma_{2}ln{PCS}_{t-1}+\gamma_{3}ln{EU}_{t-1}+\gamma_{4}ln{FD}_{t-1}+\gamma_{5}ln{GX}_{t-1}+\gamma_{D}Dummy+\mu_{t}$$ (4)

$$\Delta ln{PCS}_{t}=\beta_{0}+\sum_{i=0}^{q1}\beta_{1}{\Delta lnCE}_{t-i}+\sum_{i=0}^{q2}\beta_{2}{\Delta lnPC}_{t-i}+\sum_{i=1}^{q3}\beta_{3}{\Delta lnPCS}_{t-i}+\sum_{i=0}^{q4}\beta_{4}{\Delta lnEU}_{t-i}+\sum_{i=0}^{q5}\beta_{5}{\Delta lnFD}_{t-i}+\sum_{i=0}^{q4}\beta_{6}\Delta {lnGX}_{t-i}+\gamma_{1}ln{CE}_{t-1}+\gamma_{2}ln{PC}_{t-1}+\gamma_{2}ln{PCS}_{t-1}+\gamma_{3}ln{EU}_{t-1}+\gamma_{4}ln{FD}_{t-1}+\gamma_{5}ln{GX}_{t-1}+\gamma_{D}Dummy+\mu_{t}$$ (5)

$$\Delta ln{EU}_{t}=\beta_{0}+\sum_{i=0}^{q1}\beta_{1}{\Delta lnCE}_{t-i}+\sum_{i=0}^{q2}\beta_{2}{\Delta lnPC}_{t-i}+\sum_{i=0}^{q3}\beta_{3}{\Delta lnPCS}_{t-i}+\sum_{i=1}^{q4}\beta_{4}{\Delta lnEU}_{t-i}+\sum_{i=0}^{q5}\beta_{5}{\Delta lnFD}_{t-i}+\sum_{i=0}^{q4}\beta_{6}\Delta {lnGX}_{t-i}+\gamma_{1}ln{CE}_{t-1}+\gamma_{2}ln{PC}_{t-1}+\gamma_{2}ln{PCS}_{t-1}+\gamma_{3}ln{EU}_{t-1}+\gamma_{4}ln{FD}_{t-1}+\gamma_{5}ln{GX}_{t-1}+\gamma_{D}Dummy+\mu_{t}$$ (6)

$$\Delta ln{FD}_{t}=\beta_{0}+\sum_{i=0}^{q1}\beta_{1}{\Delta lnCE}_{t-i}+\sum_{i=0}^{q2}\beta_{2}{\Delta lnPC}_{t-i}+\sum_{i=0}^{q3}\beta_{3}{\Delta lnPCS}_{t-i}+\sum_{i=0}^{q4}\beta_{4}{\Delta lnEU}_{t-i}+\sum_{i=1}^{q5}\beta_{5}{\Delta lnFD}_{t-i}+\sum_{i=0}^{q4}\beta_{6}\Delta {lnGX}_{t-i}+\gamma_{1}ln{CE}_{t-1}+\gamma_{2}ln{PC}_{t-1}+\gamma_{2}ln{PCS}_{t-1}+\gamma_{3}ln{EU}_{t-1}+\gamma_{4}ln{FD}_{t-1}+\gamma_{5}ln{GX}_{t-1}+\gamma_{D}Dummy+\mu_{t}$$ (7)

$$\Delta ln{GX}_{t}=\beta_{0}+\sum_{i=0}^{q1}\beta_{1}{\Delta lnCE}_{t-i}+\sum_{i=0}^{q2}\beta_{2}{\Delta lnPC}_{t-i}+\sum_{i=0}^{q3}\beta_{3}{\Delta lnPCS}_{t-i}+\sum_{i=0}^{q4}\beta_{4}{\Delta lnEU}_{t-i}+\sum_{i=0}^{q5}\beta_{5}{\Delta lnFD}_{t-i}+\sum_{i=1}^{q4}\beta_{6}\Delta {lnGX}_{t-i}+\gamma_{1}ln{CE}_{t-1}+\gamma_{2}ln{PC}_{t-1}+\gamma_{2}ln{PCS}_{t-1}+\gamma_{3}ln{EU}_{t-1}+\gamma_{4}ln{FD}_{t-1}+\gamma_{5}ln{GX}_{t-1}+\gamma_{D}Dummy+\mu_{t}$$ (8)

Δ and μ_*t*_ are the difference operator and error term, respectively. Once the long run relationship among the variables is confirmed, the short run and long run causality among the variables will be determined through a vector error correction model (VECM).

## Result interpretation and discussion

The order of integration of the variables in the presence of an unknown structural break is presented in Table 1. The results of Zivot and Andrew’s structural break unit root describes that all variables are integrated as an order of one [I (1)], except for the carbon emissions per capita, which is integrated as an order of zero [I (0)]. Thus, the variables of model (1) are a mixture of [I (1)] and [I (0)], which fulfils the requirement that one can apply the ARDL model for cointegration instead of conventional cointegration tests, for instance, the Johansen cointegration. The other point that also validates the application of ARDL is the presence of a structural break.

**Table 1:** Order of integration with a structural break.

Variable	Test Stat.	Structure Break	Variable	Test Stat.	Structure Break
*lnCE*	-4.19	2003^b^	Δ*(lnCE)*	-6.92^a^	1981
*lnPC*	-3.96	2003	Δ*(lnPC)*	-5.09^a^	1981
*lnPCS*	-3.96	2003	Δ*(lnPCS)*	-5.08^a^	1981
*lnEU*	-3.19	2007	Δ*(lnEU)*	-12.72^a^	1986
*lnFD*	-2.77	2003	Δ*(lnFD)*	-5.94^a^	1995
*lnGX*	-3.98	1994	Δ*(lnGX)*	-8.16^a^	1999

^a^ and ^b^ show significance at the 1 and 5% levels, respectively.

Once the order of integration is verified for the variables in the presence of an unknown structural break, it is confirmed based on the results of Zivot and Andrew’s structural break unit root test that none of the variables are integrated higher than an order of [I (1)]. Then, we can move on to the results of the ARDL bounds test of cointegration to determine the long run relationship among the variables. The results of cointegration are portrayed in Part (A) of Table 2 where critical value bounds are given in Part (B) of Table 2. There are five cointegration vectors among the variables of this study; therefore, it is testified that the variables of the study are in a long run relationship.

**Table 2:** Results of the ARDL bounds test.

Part A				
Eq.	Model	Selected ARDL Model	F-stat.	Cointegrated
3	*lnCE/f(lnPC*,*lnPCS*,*lnEU*,*lnFD*,*lnGX)*	(1, 0, 0, 0, 1, 0)	3.99^b^	Yes
4	*lnPC/f(lnCE*,*lnPCS*,*lnEU*,*lnFD*,*lnGX)*	(1, 1, 1, 0, 0, 1)	9.81^a^	Yes
5	*lnPCS/f(lnCE*,*lnPC*,*lnEU*,*lnFD*,*lnGX)*	(1, 1, 1, 0, 0, 1)	9.72^a^	Yes
6	*lnEU/f(lnCE*,*lnPC*,*lnPCS*,*lnFD*,*lnGX)*	(1, 0, 0, 0, 0, 0)	1.11	No
7	*lnFD/f(lnCE*,*lnPC*,*lnPCS*,*lnEU*,*lnGX)*	(1, 0, 1, 0, 1, 1)	3.68^c^	Yes
8	*lnGX/f(lnCE*,*lnPC*,*lnPCS*,*lnPCS*,*lnFD)*	(1, 0, 0, 1, 0, 1)	3.61^c^	Yes
Part B				
	Critical Value Bounds			
	Lower Bound	Upper Bound		Significance
	2.26	3.35		10%
	2.62	3.79		5%
	2.96	4.18		2.5%
	3.41	4.68		1%

^a^, ^b^, and ^c^ show significance at the 1, 5, and 10% levels, respectively.

After the cointegration, the long run results are obtained using ARDL in the presence of a structural break. Long run estimates are provided in Part (A) of Table 3. The dummy represents the corresponding structural break (year). The positive and significant effect of the income per capita is highlighting that Venezuela is degrading the environmental quality. The carbon emission per capita in Venezuela is above the World emission per capita per USD 1000 of gross domestic product (GDP). This explains the inefficiency of Venezuelan economy to obtain output with less carbon emissions and shows the lack of policies and measures aimed at curbing emissions. Moreover, there is lack of measures for compensating environmental degradation in Venezuela. These results verified that the EKC hypothesis is accurate in the long run in Venezuela since the coefficients of income per capita and its squares carry the expected sign and are significant. These findings are in line with those of Nasir and Rehman [2] in the case of Pakistan. Conversely, these findings are not consistent with those of Al-mulali [40] for Cambodia and Haq et al. [3] for Morocco. Thus, it can be deduced from these results that the EKC hypothesis depends not only on per capita income but also on the overall structure of the economy; when we compare the highest real per capita income of Pakistan described in Nasir and Rehaman’s [2] study to our data, it is much lower than the real per capita income of Venezuela. In the case of this study, the lowest real per capita income is 5000 USD in 1971 whereas the highest real per capita income is 7800 USD in 2013. Thus, we can deduce from these results and discussion that the income level in Venezuela has crossed the point after which higher per capita income is not followed by an increase in per capita emissions. However, Venezuela is emitting carbon emissions per capita above regional average because of intensive oil production and consumption.

**Table 3:** Long run and short run estimates.

Part A: Long run			
Variable	Coefficient	Std. Error	t-Statistic
*lnPC*	61.067888^c^	31.293778	1.951439
*lnPCS*	-3.236433^c^	1.649521	-1.962045
*lnEU*	0.350060^b^	0.129864	2.695590
*lnFD*	-0.075564^a^	0.027288	-2.769137
*lnGX*	0.354231^a^	0.097590	3.629810
*Dummy*	0.237087	0.158567	1.495191
*Constant*	-288.261997^c^	148.099285	-1.946410
Part B: Short run			
Variable	Coefficient	Std. Error	t-Statistic
Δ*(lnPC)*	50.011414^c^	25.760687	1.941385
Δ*(lnPCS)*	-2.650470^c^	1.359147	-1.950098
Δ*(lnEU)*	0.286681^b^	0.113134	2.533985
Δ*(lnFD)*	-0.285397^a^	0.086319	-3.306321
Δ*(lnGX)*	0.290097^a^	0.076011	3.816533
*Dummy*	0.194162	0.131059	1.481486
*CointEq(-1)*	-0.818948^a^	0.149411	-5.481168
Part C: Diagnostic tests			
Test		F-stat.	Prob.
Serial correlation		0.01	0.96
Heteroskedasticity		0.48	0.86
Ramsey RESET		1.84	0.18
Jarque-Berra		1.15	0.56

^a^, ^b^, and ^c^ show significance at the 1, 5, and 10% levels, respectively.

The long run estimates show that the coefficient of energy use is significantly positive, and a ten percent increase in energy use will stimulate carbon emissions by three and a half percent. This finding is line with the related literature. Haq et al. [3] and Gamage et al. [4] also found that the energy had a positive effect on carbon emissions in the cases of Morocco and Sri Lanka. Fossil fuel consumption not only is a vital factor contributing to carbon emissions but also contributes to toxin and global warming emissions. Even the waste products of fossil fuel consumption are hazardous to health and the environment. The share of fossil fuels out of total energy use in Venezuela in 2014 was 56%. On the other hand, the share of sustainable (renewable) energy was only 15% in 2014. If one compares the emissions from fossil fuels to those of renewable energy sources, then it would be easy to understand that renewable energy resources are environmentally friendly. For instance, coal combustion in electricity generation emits between 1.4 and 3.6 pounds of CO2E/kWh, while electricity generation from wind only produces between 0.02 and 0.04 CO2E/kWh. Similarly, hydroelectric energy generation produces only between 0.1 and 0.5 CO2E/kWh. However, hydroelectric energy production has been on the decline in Venezuela since the first decade of this century due to the government policy of providing an alternative to ageing hydropower infrastructure. However, the alternative sources will further aggravate environmental degradation in Venezuela because the small power plants are fuelled with diesel or gas. Thus, it is recommended that the Venezuelan government should take initiatives to invest in hydropower to maintain and expand its capacity.

One of the reasons of energy consumption pulling the environmental degradation is lack of energy saving plans in Venezuela as government is subsidizing the fuel price which is lowest in the world. The fuel price is not covering even the cost of production in Venezuela. As a consequence the fuel efficiency in transport is less and more carbon emissions are released for every kilometer compare to countries where there is fuel efficient vehicles on the roads. Moreover, diesel and gasoline are the main sources of fuelling transport in Venezuela, while gas usage is negligible in this sector. One can count the gas stations in Venezuela on their fingertips. Therefore, it is advised that the government encourage the construction of gas stations around the country in order to reduce the use of diesel and gasoline in transport. Similarly, on the production side, the oil exploration in Venezuela is done with old extractive technology instead of modern and up to date technology that could lead to less carbon emissions. Moreover, Venezuela is fulfilling the electricity demand by building thermal plants that are running on liquid fuels instead of using natural gas which could produce same capacity of electricity by emitting less carbon emissions. Bautista [41] argued that Venezuela has all the potential to achieve sustainable development in the power sector, to reduce carbon emissions and to enhance energy efficiency. However, the result of the study at hand shows that Venezuela has yet to utilize its sustainable energy resources. Thus, the Venezuelan government has to ensure energy efficiency and enhance energy generation from sustainable and renewable resources in order to reduce carbon emissions in the future. Adewuyi and Awodumi [42] opined that policy makers should take into consideration the pollution effect of energy-growth nexus for sustainable development.

The long run coefficient of financial development carries a negative sign and is significant, which can be interpreted as the financial development in Venezuela hindering carbon emissions in the atmosphere. Khan et al. [43] and Ziaei [44] also present the ratio of private credit to GDP as a proxy for financial development; however, in both cases, the results of these studies demonstrate a positive association of financial development with carbon emissions. Zhang [20] finds that financial development stimulates carbon emissions. Similarly, Işik, Kasimati, and Ongan [45] also concluded that financial development leads to carbon emissions in Greece. This study’s findings resemble those of Jalil and Feridun [18] and Al-Mulali et al. [46] who find that financial development improves the quality of the environment. A sound financial development may decrease financing costs and channel financial resources into new technology, thus enhancing energy efficiency and, in turn, reducing carbon emissions [17, 20]. Financial institutions in Venezuela can help to develop the concept of sustainable energy in the country. The Venezuelan government can reduce carbon emissions through the further development of the financial sector and policies, such as providing soft loans to those who can deploy energy efficient technology and to renewable energy resource projects since renewable energy resources emit fewer environmentally harmful emissions.

The coefficient of government expenditure is significantly positive, and a ten percent increase in government expenditure will enhance carbon emissions by 3.5 percent. This result is in line with the argument put forward by Bernauer and Koubi [22] that government expenditure deteriorates the quality of the environment, and its effect on the environment does not depend on the quality of the government. Similarly, Lopez et al. [24] summarize that increases in government expenditure will negatively affect the environment unless the expenditure is shifted toward social and public goods, which would result in lower pollution. On the other hand, Lopez and Palacios [26] find a negative effect of government expenditure on environmental degradation. This finding then disproves the finding of Halkos and Paizanos [25] that government expenditure has no significant effect on carbon emissions. The effect of government expenditure can be explained by scale, composition, and technique effects. The scale effect postulates that government expenditure will put pressure on the environment as it enhances economic activity and hence, will harm the quality of the environment. On the other hand, government expenditure, by changing the composition of the economy, would positively affect the environment since it would generate intense human capital instead of physical capital that would negatively affect the environment. Similarly, the technical effect of government expenditure will also improve the quality of the environment since it would enhance labour efficiency and income level. However, the findings of this study show that the scale effect is prevailing in Venezuela and increases in government expenditure will deteriorate the quality of the environment. The positive effect of government expenditure on carbon emissions indicates that the Venezuelan government is not taking the effects of its expenditure on a sustainable environment into account. The government should take the lead for sustainable economic growth and environmental quality. Similarly, it is advised that the government increase its fixed capital share in renewable energy resources.

The short run results resemble the long run results in the sense that all the explanatory variables carry the same sign and are significant. The short run results are given in Part (B) of Table 3. The short run results also postulate the presence of the EKC hypothesis in Venezuela. These findings differ from those of Nasir and Rehman [2], who only confirmed the EKC hypothesis in the long run and concluded that EKC does not exist in the short run in Pakistan. Energy use is aggravating environmental degradation not only in the long run but also in the short run. The effect of financial development is also similar to the long run scenario because an improvement in financial development will reduce carbon emissions in the short run. The impact of government expenditure on carbon emissions is significantly positive; therefore, government expenditure is deteriorating the quality of the environment not only in the long run but also in the short run. The model of the study is in equilibrium, as the error correction term is significant with a negative sign. Further, the magnitude of the error correction term suggests that the model of the study will correct itself from any external shock within two years. Diagnostic tests are also conducted and the results are provided in Part (C) of Table 3. These results indicate that the error term is normally distributed and the functional form of the model is correct, as indicated by Jarque-Berra and the Ramsey RESET test, respectively. Moreover, the model does not contain econometric problems, such as serial correlation and heteroskedasticity.

Short run and long run causality results are presented in Table 4. In the short run, there is evidence of unidirectional causality from carbon emissions to economic growth, energy use, and financial development. Similarly, unidirectional causality exists from energy use to economic growth, from government expenditure to energy use and from economic growth to financial development, while there is two-way causation between financial development and energy use in the short run. In the long run, two-way causation is found between carbon emissions and economic growth, while energy use, financial development, and government expenditure are greater, causing carbon emissions. Further, unidirectional causality shifts from energy use and government expenditure to economic growth in the long run. Similarly, one-way causation is found from financial development to carbon emissions in the long run and this finding of the study resemble with the finding of Işik, Kasimati, and Ongan [45] in case of Greece. These results indicate that energy use, financial development, and government expenditure affect carbon emissions not only directly but also indirectly through economic growth. It can be deduced from these causality results that conservative and sustainable energy policies will not affect economic growth in the short run; however, such policies should look to energy demand in the long run to sustain sound economic growth. Similarly, encouraging green and sustainable concepts in financial development and in government expenditure will not alter the economic pace in the short run, but the government has to ensure sound financial development in the long run.

**Table 4:** Causality results based on VECM.

	Short run (F-stat.)					Long run
	Δ*(lnCE)*	Δ*(lnPC)*,Δ*(lnPCS)*	Δ*(lnEU)*	Δ*(lnFD)*	Δ*(lnGX)*	ECT (t-stat.)
Δ*(lnCE)*	—	0.29	0.35	1.43	0.40	-3.38^a^
Δ*(lnPC)*, Δ(*lnPCS*)	4.98^a^	—	0.43	1.10	0.44	2.04^b^
Δ*(lnEU)*	5.98^a^	4.01^a^	—	6.87^a^	6.10^a^	0.01
Δ*(lnFD)*	4.50^a^	2.77^b^	5.13^a^	—	3.17^a^	1.35
Δ*(lnGX)*	1.49	0.14	1.88	0.21	—	1.14

^a^ and ^b^ show significance at the 1 and 5% levels, respectively.

## Conclusions

This study determines the effect of energy use, financial development, and government expenditure on environmental degradation in Venezuela. Further, this study also confirms the validity of the EKC hypothesis in Venezuela. This study covers the time series from 1971 to 2013. The unit root property of the time series is examined in the presence of a structural break. The long run relationship among the variables of the study is measured using an ARDL bounds testing approach to cointegration. The results of the ARDL show that all variables are in a long run relationship. This study finds the existence of EKC in both the short run and the long run in Venezuela. The long run estimates show that the coefficient of energy use is significantly positive, meaning that energy use is decreasing the quality of the environment. Certain facts justify this finding in Venezuela. First, the share of fossil fuel out of total energy use is more than 50% in Venezuela. Second, the electricity production from hydroelectric energy has been decreasing since the first decade of this century due to the government policy of providing an alternative to ageing hydropower infrastructure. However, the alternative will further aggravate environmental degradation in Venezuela because the small power plants are fuelled by diesel or gas. Third, diesel and gasoline are the main sources of energy fuelling the transport sector, and gas usage is negligible. Therefore, it is advised that the government encourage the construction of gas stations around the country in order to reduce the use of diesel and gasoline in transport.

The long run coefficient of financial development carries a negative sign and is significant, which can be interpreted as the financial development in Venezuela hindering carbon emissions in the atmosphere. Sound financial development may decrease financing costs and channel financial resources into new technology and thus, enhance energy efficiency. Financial institutions in Venezuela can help to develop the concept of sustainable energy in the country. The Venezuelan government can reduce carbon emissions through further development of the financial sector and by coming up with policies, such as providing soft loans to those who can deploy energy efficient technology and financing renewable resource projects. This study finds that government expenditure is increasing carbon emissions in the atmosphere not only in the long run but also in the short run. This means that regarding government expenditure, the scale effect is prevailing in Venezuela. The scale effect postulates that government expenditure will put pressure on the environment as it enhances economic activity and hence, will degrade environmental quality. The positive effect of government expenditure on carbon emissions indicates that the Venezuelan government is not taking the effects of its expenditure on a sustainable environment into account. The government should take the lead in sustainable economic growth and environmental protection.

Causality analysis reveals that bidirectional causation is found between carbon emissions and economic growth, while energy use, financial development, and government expenditure are greater, causing carbon emissions. These results indicate that energy use, financial development, and government expenditure affect carbon emissions not only directly but also indirectly through economic growth. It can be deduced from these causality results that conservative and sustainable energy policies will not affect economic growth in the short run; however, such policies should look to energy demand in the long run to sustain sound economic growth. Similarly, encouraging green and sustainable concepts in financial development and in government expenditure will not alter the economic pace in the short run, but the government has to ensure sound financial development in the long run. This study recommends that in order to understand the role of government expenditure in environmental degradation, future studies should consider the share of the government in the energy sector or, even better, proxy government expenditure with government fixed capital in the energy sector. Similarly, future studies should also consider the share of the government in transport facilities and emissions from the transport sector.
